# Aplastic Anemia: Demographic and Clinical Characteristics in Costa Rica

**DOI:** 10.7759/cureus.73403

**Published:** 2024-11-10

**Authors:** María Rodríguez-Sevilla, Kathia Valverde-Muñoz, Claudia García-Hernández, Alfredo Sanabria-Castro, Ann Echeverri-McCandless, Sebastián Rojas-Chaves

**Affiliations:** 1 Hematology Department, Hospital San Juan de Dios, Costa Rican Social Security Health Fund (CCSS), San José, CRI; 2 Hematology Department, Hospital Nacional de Niños, Costa Rican Social Security Health Fund (CCSS), San José, CRI; 3 Hematology Department, Hospital México, Costa Rican Social Security Health Fund (CCSS), San José, CRI; 4 Research Unit, Hospital San Juan de Dios, Costa Rican Social Security Health Fund (CCSS), San José, CRI; 5 Pharmacology Department, Pharmacy School, Universidad de Costa Rica, San José, CRI

**Keywords:** antithymocyte globulin, aplastic anemia, costa rica, cyclosporine, first-line treatment, hematopoietic stem cell transplantation (hsct), second-line treatment, survival rate

## Abstract

Background

Aplastic anemia (AA) is a rare and heterogeneous hematological disorder defined as pancytopenia with hypocellular bone marrow in the absence of abnormal infiltration or medullary fibrosis. Various causes of AA have been identified, such as autoimmune factors, bone marrow injuries, viral infections, and genetic disorders. The symptoms of AA are directly linked to pancytopenia and the most common are fatigue, recurrent infections, and bleeding problems. The treatment of AA varies according to the severity of the disease and includes immunosuppressive therapies and bone marrow transplantation. This study aims to identify the most relevant social, clinical, and demographic characteristics of patients with AA in Costa Rica.

Methodology

A retrospective, observational study was conducted in Costa Rica by reviewing the medical records of patients diagnosed with AA in the main hospitals of the Costa Rican Social Security Health Fund (CCSS, by its acronym in Spanish). A total of 109 patients who were evaluated between 2016 and 2018 were identified. Sociodemographic, clinical, and treatment information was collected for these patients in a database that was analyzed using statistical programs such as SPSS Statistics (version 24) and GraphPad Prism (version 8).

Results

Most patients were male (56%) with an average age of 32 years. Patients were classified according to the severity of the disease, and a higher mortality at 60 months was observed in those with very severe AA and in patients over 65 years old. The most commonly used first-line treatment was the combination of rabbit antithymocyte globulin (ATG) and cyclosporine (42.9%). Patients who required a greater number of blood transfusions had a more severe disease. Further, 46 patients requiring a second line of treatment were identified, and the most common treatment in this group was the combination of ATG with eltrombopag in 19.6% of the patients. The study results present the sociodemographic and clinical characteristics of patients with AA in Costa Rica. The lack of identification of a common external factor that may influence the development of the disease is highlighted. Treatment with rabbit ATG and cyclosporine demonstrated a good response in patients. The availability and cost of treatments are important considerations, especially in developing countries.

Conclusions

The study highlights significant progress in the understanding and treatment of AA in the Costa Rican context. The results support the efficacy of the combination of antibodies and cyclosporine as a therapeutic option. The importance of adapting treatments to the characteristics of the local population is emphasized, along with the need for further research to improve long-term outcomes.

## Introduction

Aplastic anemia (AA) is a rare and heterogeneous hematological disorder defined as pancytopenia with hypocellular bone marrow in the absence of abnormal infiltration or medullary fibrosis, generally resulting from an autoimmune medullary lesion, resulting in a loss of totipotent stem cells which leads to a decrease or cessation of hematopoiesis. At the level of peripheral blood, it manifests with pancytopenia of the three specific cellular lines (erythrocytes, leukocytes, and platelets) [1,2]. Historically, the first description of AA was made by Paul Ehrlich in 1888, and later the term “aplastic anemia” was coined thanks to the collaboration of Louis Henri Vaquez in 1904 [3,4].

This condition has an annual incidence of close to two cases per million inhabitants/year in North America and Europe and is approximately three times higher in the population of East Asia. This difference has been attributed to the use of pesticides and chemical fertilizers and a possible accumulation of these xenobiotics in the food chain [5]. The incidence of AA in Latin America is lower and has been estimated at 1.6 cases per million people per year [6]. The disorder presents a distribution close to 1:1 between men and women, and in almost all population studies, half of the cases occur in the first three decades of life. However, AA is characterized by a bimodal age distribution in its presentation, with peaks in childhood (both early and late) and senescence [7].

Among the causal processes of AA that involve the loss of hematopoietic stem cells are mechanisms such as autoimmune activity, direct injury, viral infections, and clonal or genetic disorders. A triggering agent, such as drugs, chemicals, viruses, genetic mutations, or pregnancy, can trigger the expansion of cytotoxic T cells that destroy hematopoietic stem cells. This hypothesis is widely supported by clinical observations, laboratory studies, animal models, and the ability to respond to immune suppression, demonstrating an improvement in blood counts in response to immunosuppressive treatment [8,9].

Clinically, AA is classified according to the severity of associated cytopenias [7]. Symptomatic patients may manifest fatigue and cardiopulmonary findings associated with progressive anemia; however, cases of asymptomatic patients with abnormal blood counts have been reported. Likewise, due to neutropenia, these patients may present infectious processes mainly of bacterial origin, such as urinary tract infections or respiratory infections. Patients with AA may present thrombocytopenia with mucosal bleeding, menorrhagia, ecchymoses, and visual alterations due to retinal hemorrhages. Findings such as short stature, skeletal abnormalities, and café au lait spots in children and young adults should alert health professionals to the possibility of a congenital form of AA called Fanconi anemia [9]. On the other hand, findings of leukoplakia, nail dystrophy, and abnormal skin pigmentation are characteristic of another hereditary form of AA called congenital dyskeratosis [9].

In the Central American region, research on AA is scarce, and at the national level, there is only one report on pediatric patients [10]. However, epidemiological studies suggest that the incidence of AA is low, which could be associated with underreporting of this condition in selected regions. Despite this, the regional information collected is consistent with what has been reported worldwide, where an increased risk of developing AA due to frequent exposure to benzene and its derivatives is described, as well as a scarce association with specific medications [6]. The limited availability and high cost of antithymocyte globulin (ATG), either horse or rabbit, makes the treatment of acquired AA in non-candidates for allogeneic transplantation in developing and low-income countries an aspect to consider [11]. Therefore, determining the main sociodemographic, clinical, treatment, and response characteristics of patients with AA in Costa Rica is highly relevant for better understanding the population with a diagnosis of AA and improving patient management.

## Materials and methods

Following approval by the Central Scientific Ethics Committee of the Costa Rican Social Security Health Fund (CCSS) (approval number: R019-SABI-00237), a retrospective observational study was conducted in the Hematology Services or Units of the main reference hospitals between 2016 and 2018. The study was conducted in accordance with the Declaration of Helsinki’s principles and ethical standards, as well as with Costa Rica’s Research Law and our institution’s (CCSS) ethical research guidelines.

The study involved three national tertiary care hospitals for adults, i.e., Hospital San Juan de Dios, Hospital Dr. Rafael Ángel Calderón Guardia, and Hospital México; two regional secondary care hospitals for adults, i.e., Hospital Dr. Maximiliano Peralta Jiménez and Hospital San Vicente de Paul; and the National Children’s Hospital, Hospital Dr. Carlos Sáenz Herrera.

Clinical data was obtained through a review of electronic medical records from the Unique Digital Health Record system of CCSS. The collected data were entered into an electronic spreadsheet, ensuring that no personal identifiers were included. For the pediatric population, patients initially diagnosed and treated at the National Children’s Hospital but later followed up in adult hospitals were included only in the pediatric dataset. To prevent duplication, these patients were excluded from the adult hospital data.

This analysis included patients diagnosed with AA who had at least one hematological consultation between 2016 and 2018 and met the study’s eligibility criteria. To confirm the diagnosis, inclusion criteria required laboratory tests, bone marrow aspirate, and biopsy results at the time of diagnosis showing hypocellular bone marrow (<30%) with no fibrosis or abnormal infiltration. Additionally, patients needed to present with at least two of the following cytopenias: hemoglobin <10 g/dL, neutrophils <1,500/μL, and platelets <50,000/μL

Statistical analysis was conducted using SPSS Statistics for Windows (version 24, IBM Corp., Armonk, NY, USA). Continuous variables were reported as means, while categorical variables were presented as frequencies and percentages. Kaplan-Meier survival curves were used to estimate overall survival, and differences between the curves were assessed using the log-rank test. For all assessments, an alpha of 0.05 was used as the cutoff for significance.

## Results

A total of 109 patients who were being followed up with a previous AA diagnosis and met the eligibility criteria were included. Basic sociodemographic characteristics and participating hospitals are detailed in Table 1. The mean age at the time of diagnosis was 32.6 years, with a range from 0 to 83 years. The male-to-female ratio was 1.3:1.

**Table 1:** Main sociodemographic characteristics and participating hospitals. *: Includes all patients who were diagnosed and treated during their pediatric age (under 13 years of age) at this hospital, even if their follow-up visits, at the time of enrollment in this study, occurred in an adult care hospital. The number of pediatric patients included in the study was 11.

	Cases (n = 109)
Age of diagnosis (years)	
Mean	32.6
Range	0–83
	Frequency n (%)
Age groups (years)	
<10	30 (27.5)
10–39	35 (32.1)
40–65	26 (23.9)
>65	18 (16.5)
Sex	
Male	61 (56.0)
Female	48 (44.0)
Place of residence	
Rural	56 (51.4)
Urban	53 (48.6)
Occupation	
Student	34 (31.2)
Unemployed	4 (3.7)
Active worker	24 (22.0)
Housework	5 (4.6)
Retired	24 (22.0)
Other	18 (16.5)
Center where the participant was diagnosed	
National Children’s Hospital*	35 (32.1)
National Adult Tertiary Care Hospital	71 (65.0)
Regional Adult Secondary Care Hospital	3 (2.7)

The main clinical and treatment characteristics of our study population are shown in Table 2. At the time of diagnosis, the mean hemoglobin level was 8.1 g/dL, while the average leucocyte and platelet counts were 3.7 × 10^3 ^and 70.5 × 10^3^, respectively. According to severity, 33% of patients were diagnosed with non-severe AA (NSAA), 42.2% with severe AA (SAA), and 24.8% with very severe AA (VSAA).

**Table 2:** Main clinical characteristics. NSAA: non-severe aplastic anemia; SAA: severe aplastic anemia; VSAA: very severe aplastic anemia

	Mean (range)
Hematological data at diagnosis	
Hemoglobin (g/dL)	8.1 (5.3–12.5)
Leucocytes (×10^3^)	3.7 (0.21–10.8)
Thrombocytes (×10^3^)	70.5 (2.0–450)
	Frequency, n (%)
Classification of aplastic anemia according to severity at diagnosis	
NSAA	36 (33.0)
SAA	46 (42.2)
VSAA	27 (24.8)

**Figure 1:**
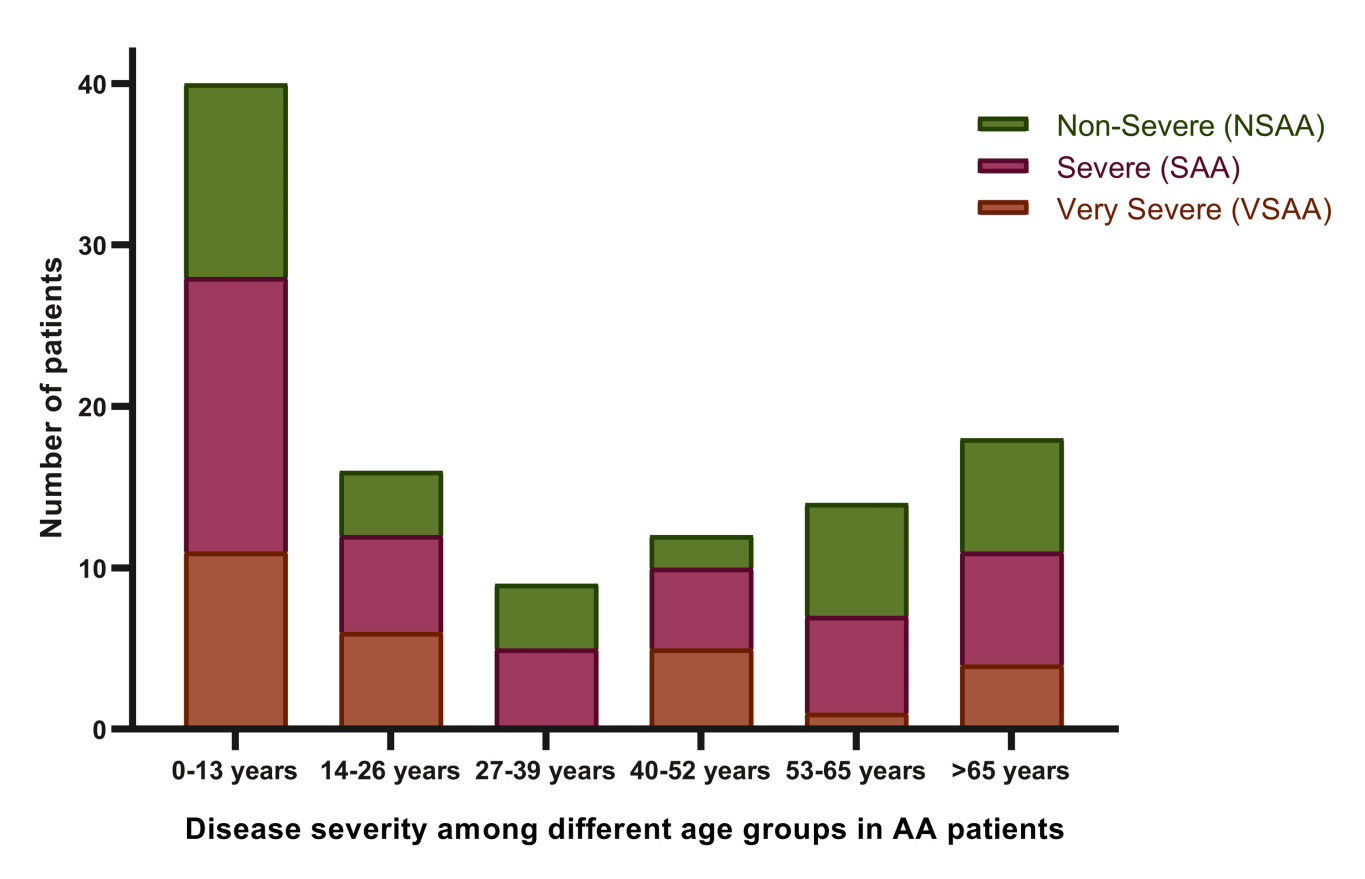
Disease severity among different age groups of AA patients. AA: aplastic anemia; NSAA: non-severe aplastic anemia; SAA: severe aplastic anemia; VSAA: very severe aplastic anemia

Disease severity varied among the different age groups, with a bimodal peak seen in all three AA severity groups between the ages of 0-13 and >65 years (Figure 1).

Cytogenetic studies were available in 68 (62.4%) patients. Among these, 66 patients had normal findings, while two patients exhibited abnormal results. Fluorescence in situ hybridization cytogenetic analysis identified one case of monosomy 7 and one case of Diamond-Blackfan anemia. No further genetic abnormalities associated with different types of AA, including Fanconi anemia, were observed in the evaluated patients.

Almost all patients, 98.2% (n = 107), received supportive care. At baseline, all patients received blood transfusions (100%, n = 107), followed by chelation therapy (1.9%, n = 2). Before receiving first-line treatment, 44.9% of patients received five or more blood transfusions (VSAA: 18.7%, n = 20; SAA: 15.9%, n = 17; and NSAA: 10.3%, n = 11; p = 0.002).

Of the 109 patients, four (3.66%) died before receiving first-line treatment. Among the 105 patients who received first-line treatment, 42.9% received the ATG rabbit-cyclosporine, while 18.1% received the ATG horse-cyclosporine regimen. A full response to first-line treatment was documented in 48.6% of the patients, regardless of the treatment prescribed. Second-line treatment was prescribed to 46 patients, of whom 19.6% received ATG (rabbit or horse) + eltrombopag, with 28.2% of patients achieving full response (Table 3).

**Table 3:** Main treatment characteristics. HSCT: hematopoietic stem cell transplantation; ATG: antithymocyte globulin

	Frequency, n (%)
First-line treatment	
HSCT	12 (11.4)
ATG rabbit-cyclosporine	45 (42.9)
ATG horse-cyclosporine	19 (18.1)
Cyclosporine-prednisone	13 (12.4)
Danazol	1 (0.9)
Unavailable	6 (5.7)
Other	9 (8.6)
First-line treatment response	
Complete response	51 (48.6)
Partial response	34 (32.3)
No response	9 (8.6)
Non-available	11 (10.5)
Patients who did not receive second-line treatment	
Died before second-line treatment	12 (11.4)
Did not require a second-line treatment	47 (44.8)
Second-line treatment	
HSCT	1 (2.2)
ATG (rabbit or horse) + eltrombopag	9 (19.6)
ATG rabbit-cyclosporine	5 (10.9)
ATG horse-cyclosporine	3 (6.5)
Cyclosporine	2 (4.3)
Cyclosporine-prednisone	5 (10.9)
Danazol	4 (8.7)
Other	17 (36.9)
Follow-up post second-line treatment	
Alive with complete response	13 (28.2)
Alive with partial response	13 (28.2)
Alive in relapse	7 (15.2)
Died after second-line treatment	13 (28.2)

First-line treatment response was recorded in 94 patients (data for 11 patients was not available). The rabbit ATG-cyclosporine regimen was the most widely used in 44 patients, followed by 17 patients who received horse ATG-cyclosporine, 12 patients who received cyclosporine-prednisone regimen, and 12 patients who underwent hematopoietic stem cell transplantation. Specifically, complete response to treatment was observed in 66.7% of patients who underwent hematopoietic stem cell transplantation, 58.8% of patients receiving horse ATG-cyclosporine, and 56.8% of patients who received rabbit ATG-cyclosporine regimen (Table 4).

**Table 4:** First-line treatment response. HSCT: hematopoietic stem cell transplantation; ATG: antithymocyte globulin

	Complete response, n(%)	Partial response, n (%)	No response, n(%)	Total number of patients
HSCT	8 (66.7)	4 (33.3)	0	12
ATG rabbit-cyclosporine	25 (56.8)	18 (40.9)	1 (2.3)	44
ATG horse-cyclosporine	10 (58.8)	3 (17.6)	4 (23.5)	17
Cyclosporine-prednisone	7 (58.3)	4 (33.3)	1 (8.3)	12
Danazol	0	1 (100)	0	1
Other	2 (25.0)	4 (50.0)	2 (25.0)	8
Total	52 (55.3)	34 (36.1)	8 (8.5)	94

**Figure 2:**
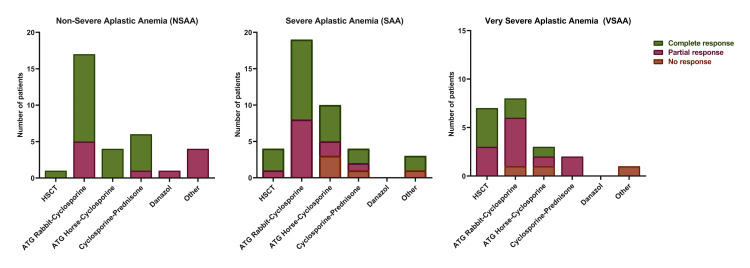
First-line treatment response according to AA severity. AA: aplastic anemia; NSAA: non-severe aplastic anemia; SAA: severe aplastic anemia; VSAA: very severe aplastic anemia

Additionally, an association between AA severity and the patient response to first-line treatment was demonstrated (p = 0.024) (Figure 2). In patients with NSAA, 11 (33.3%) exhibited a partial response, while 22 (66.7%) achieved a complete response. For those with SAA, five (12.8%) showed no response, 12 (30.8%) had a partial response, and 22 (56.4%) achieved a complete response. Among patients with VSAA, four (18.2%) had no response, 11 (50.0%) had a partial response, and seven (31.8%) achieved a complete response.

Of the 105 patients who received first-line treatment, 12 (11.4%) died, 47 (44.8%) did not require a second-line regimen, and 46 (43.8%) required a second-line treatment due to either initial therapeutic failure (partial response or no response to treatment) or subsequent relapse. Among these patients, seven (15.2%) were classified at the time of diagnosis as NSAA, 23 (50%) as SAA, and 16 (34.8%) as VSAA. The treatment regimen indicated in this population is described in Table 5.

**Table 5:** Second-line treatment response. HSCT: hematopoietic stem cell transplantation; ATG: antithymocyte globulin

	Complete response, n (%)	Partial response, n (%)	No response, n(%)	Total number of patients
HSCT	0	1 (100)	0	1
ATG rabbit/horse-cyclosporine-eltrombopag	0	8 (88.9)	1 (11.1)	9
ATG rabbit-cyclosporine	1 (20.0)	3 (60.0)	1 (20.0)	5
ATG horse-cyclosporine	3 (100)	0	0	3
Cyclosporine alone	1 (50.0)	1 (50.0)	0	2
Cyclosporine with prednisone	2 (40.0)	1 (20.0)	2 (40.0)	5
Danazol	1 (25.0)	3 (75.0)	0	4
Other	2 (11.8)	11 (64.7)	4 (23.5)	17
Total	10 (21.7)	28 (60.9)	8 (17.4)	46

In this study, a total of 13 patients received a bone marrow transplant, 12 patients as first-line treatment and one patient as second-line treatment. Donor types were matched for six patients, consisting of five related donors and one unrelated donor. Additionally, six patients had mismatched donors. Donor information for one patient is currently unavailable. The average age of these patients was 18.62 with a range between 0 and 46 years. According to severity, 7.7% of patients had a diagnosis of NSAA, 38.5% had a diagnosis of SAA, and 53.8% were diagnosed with VSAA. Overall, 53.8% of patients received between 11 and 20 blood transfusions before the hematopoietic stem cell transplantation. The survival rate following the bone marrow transplant was 100% at six months and 69.7% at 60 months (Table 6).

**Table 6:** Main clinical characteristics of patients who received a bone marrow transplant. NSAA: non-severe aplastic anemia; SAA: severe aplastic anemia; VSAA: very severe aplastic anemia

	Frequency, n(%)
Sex	
Male	7 (53.8)
Female	6 (46.2)
Place of residence	
Rural	8 (61.5)
Urban	5 (38.5)
Classification of aplastic anemia according to severity at diagnosis	
NSAA	1 (7.7)
SAA	5 (38.5)
VSAA	7 (53.8)
Blood transfusion before bone marrow transplant	
<5	2 (15.4)
5–10	1 (7.7)
11–20	7 (53.8)
>20	3 (23.1)
Bone marrow transplant response	
Complete response	8 (61.5)
Partial response	5 (38.5)
Survival rate at 6 and 60 months	
6	13 (100)
60	9 (69.2)

**Figure 3:**
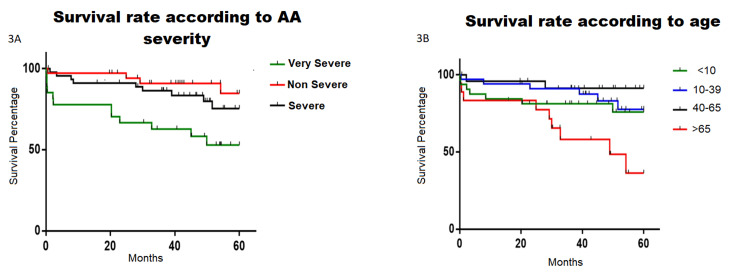
Survival curves according to (A) AA severity and (B) age groups. AA: aplastic anemia

When analyzing the Kaplan-Meier survival curves according to disease severity, patients with VSAA exhibited significantly higher mortality rates at 60 months from diagnosis compared to those with lower severity (log-rank test, p = 0.0054) (Figure 3A). Similarly, patients older than 65 years also reported higher mortality at 60 months (log-rank test, p = 0.0031) (Figure 3B). Aspects such as the place of residence and the participant’s sex did not show significant differences concerning survival at 60 months.

The distribution of deaths among the groups was notable: 12 (41.4%) patients belonged to the VSAA group, 11 (37.9%) to the SAA group, and six (20.7%) to the NSAA group. The primary cause of death across all severity groups was infection, accounting for 68.9% (n = 20) of fatalities. A diagnosis of acute myeloid leukemia was the second most common cause, contributing to 10.3% (n = 3) of deaths. The time from the start of AA therapy to the onset of leukemia in these three patients was 7, 22, and 47 months, respectively. All three patients exhibited clonal evolution; however, specific details regarding the associated cytogenetic abnormalities were lacking in the patients’ medical charts. Additionally, bleeding episodes, which occurred exclusively in the VSAA group, led to 6.9% (n = 2) of fatalities.

## Discussion

To our knowledge, this is one of the first comprehensive studies to describe the epidemiology of AA within the Costa Rican pediatric and adult population. Our research included AA patients who received at least one hematological consultation between 2016 and 2018 at the main hospitals with Hematology Services or Units of the CCSS.

In this study, the average age at diagnosis was 32.6 years, with a bimodal age distribution peaks at 0-13 and over 65 years. These findings are consistent with previous studies among Western populations, which also identified a bimodal age distribution; nevertheless, the peaks reported by Ahmed et al. were noted at 15-29 years and above 60 years [12]. Studies from Latin America, including Brazil, Argentina, and Mexico, show a bimodal age distribution with peaks ranging between 16-30 years and 31-50 years [6]. In contrast, research from Pakistan reported unimodal distribution with the highest incidence observed between 15 and 30 years [13]. Similarly, studies from Thailand showed unimodal distribution with a peak incidence between 15 and 24 years [5].

Data regarding sex distribution varies in published literature. Ahmed et al. reported a male preponderance with a 3:1 ratio in their Pakistani case report series. Our study identified a male-to-female ratio of 1.3:1, which is consistent with previous findings reported from Nigeria and India [14,15].

It is important to note that this study did not identify common risk factors associated with the development of acquired AA, which could be related to a lack of information in the medical records, and the considerable heterogenicity pertaining to sociodemographic characteristics among AA patients [12,16]. A trend observed in this study was a higher prevalence in patients residing in urban areas within provinces of the Central Valley. This may reflect the region’s higher population density, which constitutes 73.5% of Costa Rica’s population. This is compared with findings from Zimbabwe, where the majority of the patients resided in urban areas [17]. Contrary to what has been reported in other regions such as the Middle East, where illiteracy and living in rural areas have been identified as sociodemographic risk factors for AA, however, no such relationships were found in this study [16].

Treatment of AA is indeed a challenge in developing countries [13]. It varies according to the severity of the disease, the patient’s age, donor availability, and the necessity for supportive care [18]. Consistent with findings from a study conducted in China [19], in this study, almost all patients received supportive treatment (98.2%). Those who required a higher number of blood transfusions before receiving first-line treatment generally had a more severe form of the disease. This suggests that the need for transfusions is closely linked to disease severity, with more severe cases exhibiting lower hemoglobin, leucocyte, and platelet counts compared to those with less severe forms of the condition [20].

Immunosuppressive therapy with ATG is a cornerstone in the management of AA [21]. The British Committee for Standards in Haematology guidelines recommended ATG horse-cyclosporine as the first-line treatment regimen [1,19], while the China Aplastic Anemia Committee recommended ATG rabbit as a first-line immunosuppressive therapy [19]. Although there is still controversy concerning the efficacy of ATG rabbit-cyclosporine compared to ATG horse-cyclosporine as first-line [22], clinical experience and research commonly favor horse ATG due to its efficacy and a lower incidence of serum sickness, making it a preferred option in many cases [23]. However, practical factors such as availability and cost significantly influence the choice of ATG, especially in resource-limited settings of developing countries, where hematologists continuously face challenges to present cost-effective yet effective treatment strategies that ensure broader accessibility [19,22,24]. In our study, 42.9% of patients received ATG rabbit-cyclosporine as first-line treatment compared to 18.1% who were treated with ATG horse-cyclosporine, emphasizing the impact of resource constraints on therapy decisions. In our country, ATG rabbit has been more available than ATG horse, which may explain why ATG rabbit is prescribed more often to initiate treatment promptly.

In the case of bone marrow transplants, the patient’s age emerges as a significant determinant, with younger individuals demonstrating enhanced resilience and better outcomes attributed to physiological robustness. Conversely, older patients face escalated risks and potential complications associated with the transplant, prompting a meticulous risk-benefit analysis. The patient’s general health condition and comorbidities also weigh significantly in this decision-making process, demanding a thorough evaluation of various physiological functions. Furthermore, the accessibility of an HLA-identical donor considerably factors into the decision-making paradigm, as a matched donor is pivotal to minimizing graft-versus-host disease risk and optimizing overall transplant success. A judicious assessment and integration of age, health status, and donor compatibility are fundamental in formulating an appropriate course of action regarding hematopoietic stem cell transplantation for aplastic anemia patients, underscoring the complex interplay of patient-specific and logistical variables in the realm of hematopoietic stem cell transplantation. Continued advancements in transplantation protocols and research will further refine their considerations, propelling therapeutic strategies for AA toward enhanced efficacy and patient outcomes [1,20].

In our study, we found that the five-year survival among younger patients (up to the age of 40) was around 78%, a percentage similar to the survival rates reported from clinical trials with ATG in Europe [25,26]. In addition, there was no statistical difference in survival rates between rabbit or horse ATG treatments and other alternatives such as cyclosporine-prednisone, danazol, or hematopoietic stem cell transplantation, as primary treatment in all patients, which is in line with data published by the European Society for Blood and Marrow Transplantation [27,28]. It is noteworthy that the rate of complete and partial response to first-line treatment was quite favorable, accounting for 91% of all patients. In addition, patients aged 40 to 65 years experienced a five-year survival rate of around 90%. Together, these figures appear to be superior when compared to data from recent population-based AA studies in Asian patients, specifically Pakistan, where the five-year survival rate was around 60% and 47% in comparable patient groups [12,28]. This can be explained by our study design, in which patients included in the study had at least one hematological consultation within a specific time frame, not being able to analyze all patients diagnosed with AA.

Although our study comprehensively included all identifiable AA patients treated in the CCSS between 2016 and 2018, reflecting the prevalence and clinical data of this patient group, it has several limitations. First, some AA patients might not have been accurately identified in the patient lists from the participating hematological services, or they may have been deceased and/or lacked hematological evaluations within the study period. Second, as with any retrospective observational study, there is an inherent limitation due to missing or incomplete information in the medical records.

## Conclusions

This study provides a comprehensive analysis of AA in both the pediatric and adult Costa Rican population, identifying a mean age at diagnosis of 32.6 years and a male-to-female ratio of 1.3:1, which aligns with global patterns. Treatment outcomes were favorable, with the rabbit ATG-cyclosporine regimen being the most frequently prescribed first-line therapy. The five-year survival rate for younger patients was around 78%, matching or surpassing some international benchmarks. While hematopoietic stem cell transplantation demonstrated strong short-term survival rates, longer-term outcomes need further investigation. The study’s limitations include potential underreporting and incomplete data. Future research should aim to address these issues and explore regional variations in treatment practices.

## References

[1] Kulasekararaj A, Cavenagh J, Dokal I (2024). Guidelines for the diagnosis and management of adult aplastic anaemia: a British Society for Haematology Guideline. Br J Haematol.

[2] Scheinberg P (2018). Recent advances and long-term results of medical treatment of acquired aplastic anemia: are patients cured?. Hematol Oncol Clin North Am.

[3] Boddu PC, Kadia TM (2017). Updates on the pathophysiology and treatment of aplastic anemia: a comprehensive review. Expert Rev Hematol.

[4] Young NS (2018). Aplastic anemia. N Engl J Med.

[5] Issaragrisil S, Kaufman DW, Anderson T, Chansung K, Leaverton PE, Shapiro S, Young NS (2006). The epidemiology of aplastic anemia in Thailand. Blood.

[6] Maluf E, Hamerschlak N, Cavalcanti AB (2009). Incidence and risk factors of aplastic anemia in Latin American countries: the LATIN case-control study. Haematologica.

[7] Miano M, Dufour C (2015). The diagnosis and treatment of aplastic anemia: a review. Int J Hematol.

[8] Scheinberg P, Chen J (2013). Aplastic anemia: what have we learned from animal models and from the clinic. Semin Hematol.

[9] Killick SB, Bown N, Cavenagh J (2016). Guidelines for the diagnosis and management of adult aplastic anaemia. Br J Haematol.

[10] Rojas-Jiménez S, Valverde-Muñoz K (2020). [Aplastic anemia in the pediatric population of Costa Rica: 10-year experience]. Acta Méd Costarric.

[11] Howard SC, Wilimas JA, Flores A (2007). Treatment for children with severe aplastic anemia and sickle cell disease in low income countries in Latin America: a report on the recent meetings of the Monza International School of Pediatric Hematology/Oncology (MISPHO): part III. Pediatr Blood Cancer.

[12] Ahmed P, Chaudhry QU, Satti TM (2020). Epidemiology of aplastic anemia: a study of 1324 cases. Hematology.

[13] Ashraf S, Rashid A, Mughal Z (2022). Spectrum of aplastic anaemia; presentation, etiology and overall survival: ASpectrum of aplastic anaemia: presentation, etiology and overall survival-a tertiary care hospital experience tertiary care hospital experience: spectrum of aplastic anaemia. Pak BioMed J.

[14] Arewa OP, Akinola NO (2009). Survival in primary a plastic anaemia; experience with 20 cases from a tertiary hospital in Nigeria. Afr Health Sci.

[15] Bal AK, Bihari BB, Mohanty S (2019). Clinico-hematological profile of aplastic anemia in southern Odisha: an institutional study. Int J Med Res Prof.

[16] Syed MA, Rahman AA, Ghani A (2021). An investigation of selected socio-demographic factors with aplastic anemia in Pakistan: a case-control study. Int J Gen Med.

[17] Makaza M, Mahaman Y, Abdoul R (2023). Prevalence and risk factors of acquired aplastic anemia among men aged 15 years and above referred for hematological consultation at Parirenyatwa Group of Hospital Zimbabwe 2020-2022: a case-control study. MGM J Med Sci.

[18] Bulduk T (2023). Aplastic anemia from past to the present: a bibliometric analysis with research trends and global productivity during 1980 to 2022. Medicine (Baltimore).

[19] Zhu XF, He HL, Wang SQ (2019). Current treatment patterns of aplastic anemia in China: a prospective cohort registry study. Acta Haematol.

[20] Piekarska A, Pawelec K, Szmigielska-Kapłon A, Ussowicz M (2024). The state of the art in the treatment of severe aplastic anemia: immunotherapy and hematopoietic cell transplantation in children and adults. Front Immunol.

[21] Vaht K, Göransson M, Carlson K (2018). Low response rate to ATG-based immunosuppressive therapy in very severe aplastic anaemia - a Swedish nationwide cohort study. Eur J Haematol.

[22] Shin SH, Yoon JH, Yahng SA (2013). The efficacy of rabbit antithymocyte globulin with cyclosporine in comparison to horse antithymocyte globulin as a first-line treatment in adult patients with severe aplastic anemia: a single-center retrospective study. Ann Hematol.

[23] Hayakawa J, Kanda J, Akahoshi Y (2017). Meta-analysis of treatment with rabbit and horse antithymocyte globulin for aplastic anemia. Int J Hematol.

[24] Mishra K, Jandial A, Lad D, Prakash G, Khadwal A, Varma N, Malhotra P (2018). Cost and complications are limitations in resource-constrained settings for equine anti-thymocyte globulin. Eur J Haematol.

[25] Locasciulli A, Oneto R, Bacigalupo A (2007). Outcome of patients with acquired aplastic anemia given first line bone marrow transplantation or immunosuppressive treatment in the last decade: a report from the European Group for Blood and Marrow Transplantation (EBMT). Haematologica.

[26] Bacigalupo A, Socie' G, Lanino E (2010). Fludarabine, cyclophosphamide, antithymocyte globulin, with or without low dose total body irradiation, for alternative donor transplants, in acquired severe aplastic anemia: a retrospective study from the EBMT-SAA Working Party. Haematologica.

[27] Bacigalupo A, Giammarco S, Sica S (2016). Bone marrow transplantation versus immunosuppressive therapy in patients with acquired severe aplastic anemia. Int J Hematol.

[28] Vaht K, Göransson M, Carlson K (2017). Incidence and outcome of acquired aplastic anemia: real-world data from patients diagnosed in Sweden from 2000-2011. Haematologica.

